# Supplementary material for: Maternal Diet, Infection, and Risk of Cord Blood Inflammation in the Bangladesh Projahnmo Pregnancy Cohort
Source: Nutrients. 2021 Oct 26;13(11):3792. doi: 10.3390/nu13113792 (PMC8623045; doi:10.3390/nu13113792)
Supplement: Supplementary file 1 [file nutrients-13-03792-s001.zip › nutrients-1416016-supplementary.pdf]

## Supplementary Material

**Table S1.** Data collection timepoints

| Measurement/Questionnaire | Antenatal Care Visits | | | | Birth |
|---|---|---|---|---|---|
| | Enrollment | 24-28 wks | 32-36 wks | 38-40 wks | |
| Medical and obstetric history and risks | x | | | | |
| Household sociodemographic | x | | | | |
| Maternal anthropometrics & morbidity | x | x | x | x | |
| Food Frequency Questionnaire | | x | x | x | |
| Delivery outcomes | | | | | x |
| Umbilical cord blood collection | | | | | x |
| Newborn anthropometrics & morbidity | | | | | x |

**Table S2.** Frequency of intake of each of n = 39 FFQ items by time period of assessment

| | Mean Frequency 24-28 wks (n=250) | Mean Frequency 32-36 wks (n=246) | Comparison between mean intake frequency at two time points (T1 V T2): Paired T-Test, n = 245 | | |
|---|---|---|---|---|---|
| FFQ Food Item | Mean (SD) | Mean (SD) | Mean difference | t Value | Pr > \|t\| |
| Any kind of red meat | 0.61 (1.33) | 0.52 (1.14) | 0.09 | 1.14 | 0.254 |
| Any kind of poultry | 1.00 (1.39) | 1.36 (1.55) | -0.36 | -2.87 | 0.0044* |
| Any kind of liver | 0.22 (0.70) | 0.16 (0.58) | 0.06 | 1.06 | 0.2912 |
| Any kind of fish | 5.79 (1.96) | 5.91 (1.87) | -0.12 | -0.89 | 0.3718 |
| Egg | 0.82 (1.44) | 0.8 (1.34) | 0.02 | 0.19 | 0.8482 |
| Milk | 0.61(1.61) | 0.46 (1.36) | 0.15 | 1.37 | 0.1727 |
| Yoghurt/Curd | 0.03 (0.23) | 0.05 (0.50) | -0.02 | -0.7 | 0.4866 |
| Butter | 0.09 (0.77) | 0.12 (0.89) | -0.03 | -0.43 | 0.6671 |
| Peanuts | 0.00 (0.06) | 0.03 (0.45) | -0.03 | -0.85 | 0.3972 |
| Vegetable oil | 6.88 (0.72) | 6.9 (0.71) | -0.02 | 0.07 | 0.9481 |
| Carrots | 0.03 (0.23) | 0.00 (0.00) | 0.03 | 2.15 | 0.0322* |
| Corn | 0.03 (0.45) | 0.00 (0.00) | 0.03 | 1 | 0.3183 |
| Tomatoes (red) | 1.11(1.56) | 0.55 (1.16) | 0.56 | 5.67 | <.0001* |
| Pumpkin (yellow) | 0.64 (1.09) | 0.66 (1.09) | -0.02 | -0.14 | 0.8917 |
| Dark leafy greens | 2.9 (2.21) | 2.68 (2.18) | 0.22 | 1.98 | 0.0491* |
| Beans / Peas | 0.61(1.20) | 0.32 (0.70) | 0.29 | 4.08 | <.0001* |
| Papaya (yellow) | 0.15 (0.45) | 0.23 (0.69) | -0.08 | -1.37 | 0.1715 |
| Jackfruit | 0.86 (1.61) | 1.38 (1.90) | -0.52 | -2.87 | 0.0044* |
| Mango | 1.20 (2.20) | 1.76 (2.36) | -0.56 | -2.39 | 0.0178* |
| Banana | 0.37 (0.84) | 0.40 (0.95) | -0.03 | -0.47 | 0.6417 |

| | | | | | |
|---|---|---|---|---|---|
| Orange | 0.18 (0.71) | 0.06 (0.36) | 0.12 | 2.49 | 0.0134* |
| Watermelon | 0.29 (0.82) | 0.02 (0.15) | 0.27 | 4.86 | <.0001* |
| Melon (yellow) | 0.00 (0.00) | 0.00 (0.00) | 0 | -1 | 0.3183 |
| Guava | 0.21 (0.81) | 0.36 (1.08) | -0.15 | -1.71 | 0.0891 |
| Moringa leaves | 0.20 (1.03) | 0.04 (0.41) | 0.16 | 2.05 | 0.041* |
| Green pepper | 1.94 (2.11) | 1.60 (1.87) | 0.34 | 2.12 | 0.0354* |
| Spinach or Cassava | 0.41 (1.06) | 0.65 (1.15) | -0.24 | -2.68 | 0.0078* |
| Radish leaves | 0.01 (0.09) | 0.01 (0.11) | 0 | -0.45 | 0.6556 |
| Sweet potato | 0.12 (0.81) | 0.00 (0.06) | 0.12 | 2.1 | 0.0364* |
| Whole milk powder | 1.85 (2.46) | 2.01 (2.63) | -0.16 | -0.92 | 0.3581 |
| Khoa | 0.29 (1.25) | 0.39 (1.42) | -0.1 | -0.8 | 0.4217 |
| Cherries | 0.00 (0.00) | 0.00 (0.00) | 0 | . | . |
| Methi leaves | 0.00 (0.00) | 0.00 (0.00) | 0 | . | . |
| Bathua leaves | 0.02 (0.22) | 0.00 (0.00) | 0.02 | 1.74 | 0.0833 |
| Soyabean | 0.09 (0.65) | 0.08 (0.41) | 0.01 | 0.3 | 0.7616 |
| Lentils | 1.59 (1.54) | 1.54 (1.33) | 0.05 | 0.62 | 0.5377 |
| Bajra | 0.01 (1.19) | 0.00 (0.00) | 0.01 | -1 | 0.3183 |
| Red palm oil | 0.00 (0.00) | 0.00 (0.00) | 0 | . | . |
| Plantain | 0.15 (0.57) | 0.10 (0.38) | 0.05 | 0.98 | 0.3291 |

**Table S3.** The adjusted odds of cord blood inflammation (>75th percentile) by tertile of maternal nutrient intake (n=251 mothers and their healthy, term infants). [Reference is the middle tertile of intake]

| Nutrient[1] | Tertiles of Intake (range) | IL-1α AOR[1] [95%CI] | IL-1β AOR[1] [95%CI] | IL-6 AOR[1] [95%CI] | IL-8 AOR[1] [95%CI] | CRP AOR[1] [95%CI] | Any marker[2] AOR[1] [95%CI] |
|---|---|---|---|---|---|---|---|
| Vit A (mcg) | <33% (32.1–286) | 1.50 [0.7 - 3.21] | 2.13 [0.93 - 4.87] | 1.18 [0.56 - 2.52] | 1.76 [0.81 - 3.79] | 1.16 [0.56 - 2.41] | 1.27 [0.64 - 2.51] |
| | >66% (482–2741) | 0.96 [0.44 - 2.1] | 1.81 [0.8 - 4.1] | 0.78 [0.36 - 1.69] | 0.79 [0.35 - 1.78] | 0.62 [0.29 - 1.35] | 1.15 [0.58 - 2.28] |
| Vit B1 (mg) | <33% 0.09–0.22) | 2.13 [0.98 - 4.65] | 1.76 [0.79 - 3.95] | 2.07 [0.95 - 4.48] | **2.27 [1.05 - 4.91]** | 1.11 [0.52 - 2.34] | 1.14 [0.57 - 2.27] |
| | >66% (0.32–0.56) | 1.30 [0.59 - 2.85] | 0.75 [0.34 - 1.68] | 1.02 [0.46 - 2.26] | 0.74 [0.32 - 1.67] | 1.01 [0.48 - 2.12] | 0.91 [0.46 - 1.8] |
| Vit B2 (mg) | <33% (0.12–0.37) | **3.17 [1.39 - 7.19]** | 2.11 [0.93 - 4.8] | **2.25 [1.03 - 4.94]** | **2.45 [1.12 - 5.37]** | **2.52 [1.17 - 5.45]** | **2.84 [1.37 - 5.88]** |
| | >66% (0.55–1.60) | 1.86 [0.83 - 4.15] | 1.14 [0.52 - 2.51] | 1.10 [0.5 - 2.41] | 0.74 [0.33 - 1.68] | 1.10 [0.5 - 2.42] | 1.45 [0.74 - 2.86] |
| Vit B3 (mg) | <33% (3.55 – 7.34) | **2.30 [1.03 - 5.12]** | **2.94 [1.25 - 6.9]** | 1.60 [0.74 - 3.46] | **3.23 [1.42 - 7.31]** | 0.96 [0.46 - 2.02] | 1.70 [0.84 - 3.44] |
| | >66% (0.20–0.56) | 1.61 [0.72 - 3.58] | 1.70 [0.75 - 3.84] | 0.80 [0.36 - 1.78] | 1.32 [0.57 - 3.06] | 0.62 [0.29 - 1.34] | 1.04 [0.52 - 2.06] |
| Vit B6 (mg) | <33% (0.20–0.47) | 1.78 [0.82 - 3.87] | 1.92 [0.85 - 4.34] | **2.47 [1.12 - 5.44]** | **2.35 [1.08 - 5.13]** | 1.56 [0.74 - 3.3] | 1.43 [0.71 - 2.9] |
| | >66% (0.68–1.49) | 1.20 [0.55 - 2.61] | 0.75 [0.34 - 1.67] | 1.09 [0.49 - 2.44] | 0.71 [0.31 - 1.63] | 0.90 [0.41 - 1.95] | 0.80 [0.4 - 1.58] |
| Vit B9 (Folate) (mcg) | <33% (9.36–77.3) | 1.60 [0.73 - 3.51] | 2.04 [0.88 - 4.71] | 1.16 [0.54 - 2.47] | **2.28 [1.04 - 5.02]** | 1.36 [0.64 - 2.89] | 1.14 [0.57 - 2.3] |
| | >66% (121–321) | 1.28 [0.6 - 2.73] | 1.26 [0.57 - 2.76] | 0.55 [0.25 - 1.19] | 0.78 [0.35 - 1.76] | 0.86 [0.4 - 1.85] | 0.89 [0.45 - 1.76] |
| Vit B12 (mcg) | <33% (0.20–1.60) | 0.60 [0.28 - 1.3] | 0.49 [0.21 - 1.13] | **0.42 [0.19 - 0.92]** | 0.49 [0.23 - 1.07] | 0.75 [0.35 - 1.6] | 0.83 [0.41 - 1.67] |
| | >66% (1.89–2.75) | 0.63 [0.29 - 1.37] | 0.54 [0.24 - 1.24] | **0.38 [0.17 - 0.84]** | **0.42 [0.19 - 0.95]** | 0.71 [0.33 - 1.56] | 0.70 [0.35 - 1.4] |
| Vit C (mg) | <33% (1.50–57.3) | 1.28 [0.59 - 2.8] | 1.28 [0.57 - 2.89] | 1.31 [0.61 - 2.81] | 1.23 [0.57 - 2.62] | 0.65 [0.31 - 1.38] | 0.89 [0.45 - 1.77] |
| | >66% (101–261) | 1.18 [0.56 - 2.48] | 0.76 [0.35 - 1.65] | 0.82 [0.38 - 1.74] | 0.52 [0.24 - 1.15] | 0.52 [0.25 - 1.1] | 0.88 [0.45 - 1.73] |
| Vit D (mcg) | <33% (2.26–7.71) | 0.66 [0.29 - 1.52] | 1.44 [0.59 - 3.52] | 1.33 [0.57 - 3.07] | 1.18 [0.51 - 2.73] | 0.90 [0.39 - 2.06] | 0.84 [0.39 - 1.79] |
| | >66% (11.6–27.13) | **0.48 [0.23 - 0.99]** | 0.65 [0.31 - 1.37] | 0.80 [0.39 - 1.67] | 0.57 [0.27 - 1.2] | 0.86 [0.42 - 1.77] | **0.46 [0.23 - 0.91]** |

| | | | | | | | |
|---|---|---|---|---|---|---|---|
| Vit E (mg) | <33% (1.76–2.74) | 0.91 [0.42 - 1.98] | 1.00 [0.43 - 2.34] | 0.82 [0.38 - 1.79] | 1.06 [0.49 - 2.3] | 0.90 [0.43 - 1.9] | 0.54 [0.27 - 1.09] |
| | >66% (3.45–6.01) | 0.79 [0.38 - 1.65] | 0.76 [0.36 - 1.61] | 0.60 [0.28 - 1.26] | **0.42 [0.19 - 0.92]** | 0.56 [0.26 - 1.19] | **0.44 [0.22 - 0.89]** |
| Iron (mg) | <33% (1.05–2.76) | 1.57 [0.73 - 3.38] | **2.37 [1.03 - 5.45]** | 1.52 [0.72 - 3.25] | **2.28 [1.05 - 4.97]** | 0.94 [0.45 - 1.94] | 1.06 [0.54 - 2.09] |
| | >66% (4.20–7.14) | 1.30 [0.61 - 2.78] | 1.52 [0.68 - 3.37] | 0.96 [0.45 - 2.05] | 1.01 [0.45 - 2.24] | 0.70 [0.34 - 1.48] | 0.75 [0.38 - 1.46] |
| Selenium (mg) | <33% (0.01–0.03) | 1.43 [0.67 - 3.06] | 1.62 [0.72 - 3.66] | 1.01 [0.48 - 2.16] | 1.79 [0.84 - 3.83] | 0.95 [0.46 - 1.97] | 0.95 [0.48 - 1.89] |
| | >66% (0.04–0.08) | 1.23 [0.58 - 2.62] | 1.43 [0.66 - 3.1] | 1.01 [0.48 - 2.11] | 1.02 [0.47 - 2.23] | 0.65 [0.3 - 1.37] | 0.66 [0.33 - 1.3] |
| Zinc (mg) | <33% (1.07-2.56) | 2.10 [0.95 - 4.65] | **2.52 [1.07 - 5.91]** | 2.08 [0.93 - 4.65] | 2.20 [1 - 4.86] | 1.60 [0.73 - 3.47] | 1.75 [0.86 - 3.58] |
| | >66% (3.61–7.60) | 1.23 [0.56 - 2.7] | 1.26 [0.56 - 2.82] | 1.24 [0.57 - 2.73] | 0.68 [0.3 - 1.55] | 1.06 [0.49 - 2.28] | 0.97 [0.49 - 1.92] |
| ALA (mg) | <33% (20.0–143) | 1.48 [0.67 - 3.24] | 1.58 [0.68 - 3.69] | 1.25 [0.57 - 2.73] | 2.10 [0.95 - 4.68] | 1.35 [0.64 - 2.87] | 1.10 [0.54 - 2.22] |
| | >66% (243–575) | 1.13 [0.54 - 2.4] | 1.30 [0.61 - 2.77] | 0.99 [0.47 - 2.09] | 0.94 [0.43 - 2.06] | 0.67 [0.31 - 1.43] | 0.72 [0.37 - 1.42] |
| DHA (mg) | <33% (17.7–74.1) | 0.69 [0.33 - 1.44] | 0.95 [0.44 - 2.08] | 0.91 [0.43 - 1.93] | 1.04 [0.49 - 2.22] | 1.26 [0.61 - 2.64] | 0.90 [0.46 - 1.75] |
| | >66% (85.3–118) | 0.77 [0.36 - 1.69] | 0.97 [0.42 - 2.23] | 1.15 [0.53 - 2.51] | 1.27 [0.57 - 2.82] | 1.40 [0.63 - 3.08] | 0.83 [0.41 - 1.67] |
| DPA (mg) | <33% (2.77–30.4) | 0.89 [0.35 - 2.26] | 0.93 [0.35 - 2.43] | 0.75 [0.3 - 1.89] | 1.02 [0.39 - 2.66] | 1.76 [0.67 - 4.6] | 1.31 [0.57 - 2.97] |
| | >66% (38..7–38.7) | 1.32 [0.56 - 3.08] | 1.46 [0.61 - 3.52] | 1.08 [0.47 - 2.51] | 1.46 [0.6 - 3.53] | 1.86 [0.74 - 4.67] | 1.39 [0.64 - 2.98] |
| ETA (mg) | <33% (3.27–21.1) | 0.95 [0.44 - 2.05] | 1.06 [0.47 - 2.42] | 0.73 [0.34 - 1.58] | 1.18 [0.53 - 2.61] | 1.47 [0.68 - 3.18] | 1.57 [0.78 - 3.16] |
| | >66% (24.5–35.7) | 1.00 [0.48 - 2.1] | 1.16 [0.53 - 2.54] | 0.77 [0.37 - 1.6] | 1.35 [0.63 - 2.9] | 1.53 [0.72 - 3.26] | 1.34 [0.69 - 2.61] |
| EPA (mg) | <33% (2.26–24.8) | 0.86 [0.4 - 1.84] | 0.74 [0.33 - 1.68] | 0.97 [0.45 - 2.09] | 0.92 [0.42 - 1.99] | 1.40 [0.64 - 3.02] | 1.46 [0.73 - 2.93] |
| | >66% (31.6–31.6) | 0.89 [0.42 - 1.86] | 0.79 [0.36 - 1.72] | 0.90 [0.42 - 1.9] | 0.89 [0.42 - 1.9] | 1.59 [0.74 - 3.4] | 1.17 [0.6 - 2.28] |
| ARA (mg) | <33% (33.2–67.1) | 1.29 [0.6 - 2.75] | 0.74 [0.33 - 1.63] | 1.34 [0.64 - 2.8] | 1.77 [0.83 - 3.81] | 1.43 [0.68 - 3] | 1.23 [0.62 - 2.44] |
| | >66% (96.5–234.8) | 1.19 [0.55 - 2.58] | 0.64 [0.28 - 1.45] | 0.59 [0.27 - 1.34] | 1.04 [0.46 - 2.35] | 1.15 [0.53 - 2.49] | 0.75 [0.38 - 1.5] |
| LA (mg) | <33% (1020–1994) | 1.72 [0.81 - 3.67] | 1.73 [0.76 - 3.9] | 1.35 [0.63 - 2.9] | **2.35 [1.07 - 5.15]** | 1.47 [0.69 - 3.12] | 1.89 [0.93 - 3.86] |
| | >66% (2116–3561) | 0.78 [0.36 - 1.69] | 1.10 [0.51 - 2.38] | 0.85 [0.4 - 1.8] | 0.96 [0.43 - 2.12] | 0.87 [0.41 - 1.85] | 0.83 [0.42 - 1.64] |
| **Low Nutrient Intake Categories (<33rd percentile)[3]** | | | | | | | |
| Water-soluble vitamins[4] | | 1.08 [0.55 - 2.13] | 1.28 [0.64 - 2.57] | 1.19 [0.6 - 2.35] | 1.60 [0.79 - 3.24] | 1.40 [0.71 - 2.79] | 1.07 [0.58 - 1.97] |
| Fat-soluble vitamins[4] | | 1.33 [0.7 - 2.55] | 1.68 [0.86 - 3.27] | 1.18 [0.61 - 2.26] | **1.96 [1.01 - 3.82]** | 1.55 [0.81 - 2.95] | 1.34 [0.74 - 2.43] |
| Minerals | | 1.51 [0.8 - 2.85] | **2.12 [1.07 - 4.20]** | 1.23 [0.65 - 2.32] | **2.07 [1.07 - 3.99]** | 1.68 [0.9 - 3.15] | 1.49 [0.84 - 2.65] |
| LCPUFAs | | 1.03 [0.53 - 1.98] | 0.90 [0.45 – 1.80] | 1.99 [0.98 - 4.04] | 1.42 [0.72 - 2.83] | 1.38 [0.70 - 2.71] | 0.96 [0.53 - 1.75] |

1. Reference category for models of individual nutrient intake tertiles are levels between the $33^{rd}$-$67^{th}$ percentile of the sample distribution. Models adjusted for socioeconomic status (continuous), primiparity, maternal upper-arm circumference < 22 cm at enrollment, tobacco and/or betel nut use, season of initial antenatal assessment, and maternal educational attainment (years). Analytic sample, n = 244 dyads with both nutrient and inflammatory marker data available

2. Any marker refers to elevation (> $75^{th}$ percentile) of one or more of the five inflammatory markers examined

3. Low nutrient intake category variables are binary: operationalized as one or more deficiencies within nutrient category (at least one nutrient with value < $33^{rd}$ percentile) versus no deficiencies (reference, no values less than $33^{rd}$ percentile) within nutrient category

4. Water-soluble vitamins include all Bs and C; Fat-soluble vitamins include A, D, and E

*Statistically significant odds ratios (p < 0.05) are bolded
